# BMSC-derived exosomes protect against kidney injury through regulating klotho in 5/6 nephrectomy rats

**DOI:** 10.1186/s40001-022-00742-8

**Published:** 2022-07-11

**Authors:** Feng Wan, Ru-chun Yang, Yue-wen Tang, Xuan-li Tang, Tian Ye, Jie Zheng, Hua-qin Zhang, Yi Lin

**Affiliations:** 1 Department of Nephrology, Hangzhou TCM Hospital Affiliated to Zhejiang Chinese Medical University, Tiyuchang Road 453, Hangzhou, 31007 People’s Republic of China; 2 Key Laboratory of Kidney Disease Prevention and Control Technology, Hangzhou, Zhejiang China

**Keywords:** BMSC-derived exosome, Renoprotective, Klotho, 5/6 Nephrectomy rats

## Abstract

**Aim:**

The aim of this study was to investigate the renoprotective effects of exosomes derived from rat bone marrow mesenchymal stem cells (rBMSCs) in a rat model of 5/6 nephrectomy (Nx)-induced chronic kidney disease (CKD).

**Methods:**

A rat model of 5/6 Nx-induced CKD was established using conventional method. rBMSC-derived exosomes were isolated using ultracentrifugation and characterized. The exosomes were injected into 5/6 Nx rats through the caudal vein. After 12 weeks, 24 h proteinuria, serum creatinine (SCr), and blood urea nitrogen (BUN) levels were evaluated, and renal pathology was analyzed by H&E and Masson staining, and transmission electron microscopy. The expression of klotho was analyzed and the activity of the klotho promoter was evaluated using a luciferase reporter assay.

**Results:**

The isolated exosomes showed typical morphological features. Exosomes transplantation reduced 24 h urinary protein excretion, and SCr and BUN levels in 5/6 Nx-induced CKD rats. Furthermore, renal pathology was improved in the exosome-treated 5/6 Nx rats. Mechanistically, the exosomes significantly upregulated the activity of klotho promoter and its expression.

**Conclusions:**

Transplantation of rBMSC-derived exosomes may protect against kidney injury, probably by regulating klotho activity and expression. Our results provide a theoretical basis for the application of rBMSC-derived exosomes in CKD therapy.

**Supplementary Information:**

The online version contains supplementary material available at 10.1186/s40001-022-00742-8.

## Introduction

Chronic kidney disease (CKD) is a global public health concern, and poses a substantial risk of progression to end-stage renal disease [1]. The first-line treatment for CKD is hormonal and immunosuppressant therapy (Additional file 1). However, its clinical use is limited due to associated adverse effects and high incidence of hormone resistance [2]. Thus, there is an urgent need for a novel treatment strategy for CKD.

Mesenchymal stem cells (MSCs) are pluripotent stem cells that play important roles in treating various diseases, including CKD [3–5]. The mechanisms underlying therapeutic effects of bone marrow MSCs (BMSCs) are rather complex, with cell differentiation and paracrine signaling being part of these mechanisms [6]. As mediators of paracrine effects, exosomes carry active proteins and nucleic acids and have been proposed as alternative therapeutic agents with broad prospects (Additional file 2).

Klotho is identified as an anti-aging gene and is highly expressed in kidney [7]. α-Klotho deficiency has been reported in animal models of CKD as well as in patients with CKD [7–9]. Thus, regulating klotho expression may be a promising strategy for treating CKD.

In this current study, we explored the effects of rat BMSC (rBMSC)-derived exosomes on kidney injury and regulating klotho expression in a rat model of 5/6 nephrectomy (5/6 Nx)-induced CKD. Our study outcomes provide new insights for devising therapeutic strategies for CKD in the future (Additional file 3).

## Materials and methods

### Exosome isolation and identification

rBMSCs were isolated from the bone marrow, as reported in our previous study [10]. The rBMSCs in passage 3–5 (P3–P5) were used for exosome extraction. Exosome-free medium was used to avoid interference from bovine exosomes [11, 12]. After 48 h culture, the supernatant was collected and the exosomes were isolated by sequential centrifugation at 300×*g* for 10 min, 2000×*g* for 20 min, and 10,000×*g* for 30 min to remove cell debris. Next, the samples were centrifuged at 100,000×*g* for 3 h. Subsequently, the pellets were resuspended in PBS and passed through a 0.22 μm filter (Additional file 4). CD63 and CD9 were used as markers for exosomes identification. In addition, the size distribution and concentration of exosomes were analyzed using nanoparticle tracking analysis (NTA) by the Umibio Science and Technology Group (Shanghai). The characterized exosomes were either used immediately or stored at −80 °C until further analysis.

### Animal study

Sprague–Dawley rats were purchased from the Zhejiang Academy of Medical Sciences and housed under standard conditions, as previously described [10]. After 1 week of acclimation, the rats were subjected to 5/6 nephrectomy as reported earlier [13] and were randomly divided into three groups: control group, 5/6-nephrectomized (5/6 Nx), and exosome group. The rats were then injected with BMSC-derived exosome (1 × 10^7^/mL) through the caudal vein on days 30 and 45 (Additional file 5). At week 12, the rats were moved to metabolism cages for 24 h urine collection and urinary protein levels were quantified immediately by a biochemical analyser (HITACHI 7180) after centrifugation (Additional file 6). The rats were then sacrificed, and the blood and remnant kidney tissues were harvested for further analyses. The blood samples were centrifuged at 6000×*g*, 4 ℃ for 10 min to obtain serum and stored at −80 ℃ for further experiments.

All the procedures were approved by the Institutional Animal Care and Use Committee of the Zhejiang Academy of Medical Sciences (No. ZJCLA-IACUC-20070015).

### qRT-PCR

Total RNA was extracted from rat kidney tissues using TRIzol reagent, according to the manufacturer’s protocol. cDNA was synthesized using a reverse transcription kit (RR036A, Takara, Japan). The primer sequences were as follows: Klotho-Forward: 5′-AACAACTTTCTTCTGCCCTATTTC-3′, Klotho-Reverse: 5′-TGAGCGGTCACTAAGCGAAT-3′, α-SMA-Forward: 5′-GCGTGGCTATTCCTTCGTGACTAC-3′, α-SMA-Reverse: 5′-CCATCAGGCAGTTCGTAGCTCTTC-3′, desmin-Forward: 5′-AATGACCGCTTCGCCAACTACTTC-3′, desmin-Reverse: 5′-GCTCTCGCATCTCCTCCTCGTAG-3′, Vimentin-Forward: 5′-GTCCGTGTCCTCGTCCTCCTAC-3′, Vimentin-Reverse: 5′-TAGAGGCTGCGGCTAGTGCTG-3′, GAPDH-Forward: 5′-ACCACAGTCCATGCCATCAC-3′, GAPDH-Reverse: 5′-TCCACCACCCTGTTGCTGTA-3′. The mRNA expression levels were normalized to that of GAPDH.

### H&E and Masson staining and TEM analysis

Freshly isolated kidney tissues were fixed in 4% paraformaldehyde, embedded in paraffin, cut into 3 μm thick section, and stained with hematoxylin and eosin (H&E) and Masson’s trichrome for kidney histological analysis. A semi-quantitative evaluation of renal tissues pathology was blindly accomplished by an expert pathologist. Masson’s trichrome stained sections were used to evaluated interstitial tubular fibrosis and tubular atrophy and the scoring criteria was as follows: (0) denotes no change; grade (1) changes affecting < 25% tubular damage (mild); grade (2) changes affecting 25–50% of tubules (moderate); grade (3) changes affecting > 50% of tubules (severe). Besides, H&E stained sections were used to evaluate the degree of glomerulosclerosis and the glomerular sclerosis index (GSI) was calculated as described previously [10].

For TEM, kidney tissues of 1 mm^3^ were fixed in 2.5% glutaraldehyde for 6 h, rinsed three times in 0.1 M PBS, post-fixed with 1% osmium tetroxide for 1 h, dehydrated in graded solutions of acetone, and embedded in graded Epon 812. Subsequently, 80–100 nm sections were cut and stained with uranyl acetate and lead citrate, and observed using a JEM-1400 transmission electron microscope (JEOL, Japan).

### Immunohistochemical analysis

3 μm serial paraffin sections of the kidney were subjected to immunohistochemical staining. The sections were treated with Tris–EDTA buffer (pH 9.0) for antigen retrieval and incubated by the primary antibodies, including rabbit polyclonal anti-αSMA (14395-1-AP, ProteinTech) (1:500), rabbit monoclonal anti-desmin (ab32362, abcam) (1:500), and rabbit monoclonal anti-vimentin (ab92547, abcam) (1:100). Thereafter, slides were incubated with HRP-conjugated anti-rabbit IgG Ab at 37 °C for 1 h. Finally, the slides were visualized by DAB (brown coloration) or AEC (red coloration) for 10 min and counterstained with hematoxylin for 10 s. The staining results were obtained via light microscope.

### Luciferase reporter gene assay

HEK293T cells were co-transfected with GV238-klotho-p-Luc (klotho promotor, NM_004795, ~ 2000 bp) and pRL-TK-luc at a 50:1 ratio using lip3000 according to the manufacturer’s instructions. The transfected cells were treated with TGF-β1 (1 ng/μL), exosomes (0.4 mg), or co-cultured with BMSCs (5 × 10^4^/well) in a Transwell plate for 24 h. GW4869 (GLPBIO, Montclair, CA, USA) was used as an inhibitor of exosome biogenesis and release. The cells were then harvested and lysed to determine firefly and Renilla luciferase activities using a dual luciferase reporter gene assay (Promega). Firefly luciferase activity was normalized to that of Renilla luciferase. The experiment was conducted three times.

### Western blotting

Rat kidney tissues were lysed using RIPA buffer containing a proteinase inhibitor cocktail (Beyotime). The protein content of cell lysates was quantified using the BCA assay, separated by SDS–PAGE using 10% gels, transferred onto a nitrocellulose membrane, blocked with 5% skimmed milk, and then incubated with the primary antibodies specific for klotho (Abclonal, A12028) and GAPDH (ProteinTech, 60,004-1-Ig). Subsequently, the membranes were washed and incubated with infrared-labeled anti-rabbit/mouse IgG Ab (1:15,000). Finally, the signal was detected using an Odyssey CLx image system (LI-COR).

### Statistical analysis

Data are presented as mean ± standard deviation (SD). Graphs were constructed using the Prime GraphPad software (version 8.0). The data were analyzed using one-way analysis of variance (ANOVA), followed by Tukey’s post hoc test for multiple comparisons. Statistical significance was set at *p* < 0.05.

## Results

### Characterization of exosomes extracted from rBMSCs

**Fig. 1:**
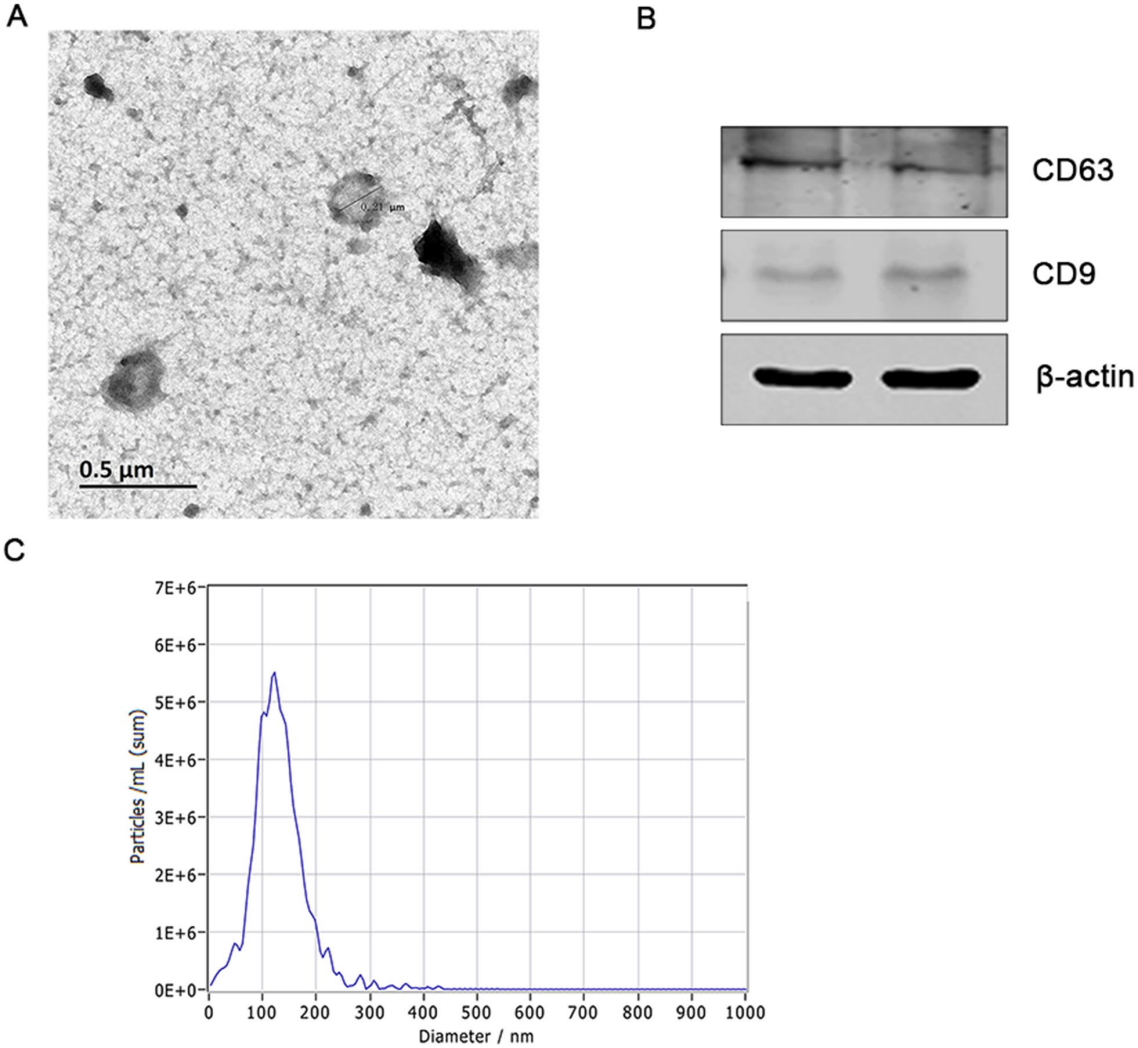
Identification of rBMSC-derived exosomes. **A** Morphological characteristics of rBMSC-derived exosomes under a transmission electron microscope. Scale bar 0.5 μm. **B** Western blot analysis was performed to identify exosomes using their specific makers CD63 and CD9. **C** Size distribution of exosomes, as determined using a ZetaView analysis system

Morphologically, rBMSC-derived exosomes appeared round with a central depression and tested positive for its specific markers, CD63 and CD9 (Fig. 1A, B). The mean diameter of exosomes was approximately 130 nm in size, as determined using a ZetaView (Fig. 1C). These results indicated that the isolated exosomes had typical characteristics of exosomes.

### rBMSCs-derived exosome improved the kidney function of 5/6 Nx rats

**Fig. 2:**
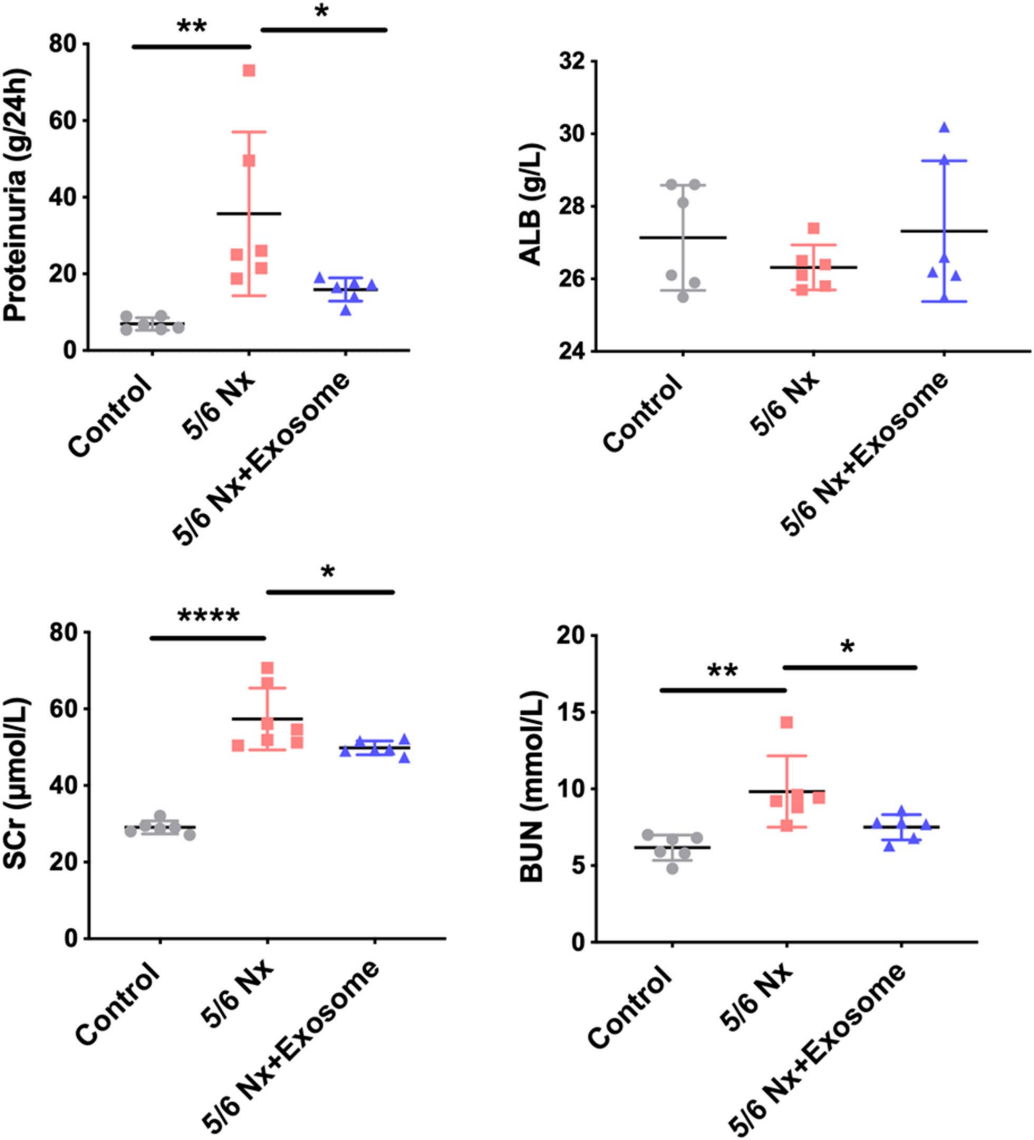
Effect of exosomes on renal function. The renal function indexes including 24 h proteinuria, serum creatinine (SCr), blood urea nitrogen (BUN), and ALB were examined by a biochemical analyzer. Data are shown as the mean ± SD. *p < 0.05, **p < 0.01, ****p < 0.0001

The 5/6 Nx rats exhibited increased 24 h proteinuria, and SCr and BUN levels compared to control rats. After exosome intervention, 24 h proteinuria and SCr and BUN levels decreased markedly compared with those in the untreated 5/6 Nx rats (Fig. 2). These data suggest that rBMSC-derived exosomes partially improved the kidney function.

### rBMSCs-derived exosome improved renal pathological injury in 5/6 Nx rats

**Fig. 3:**
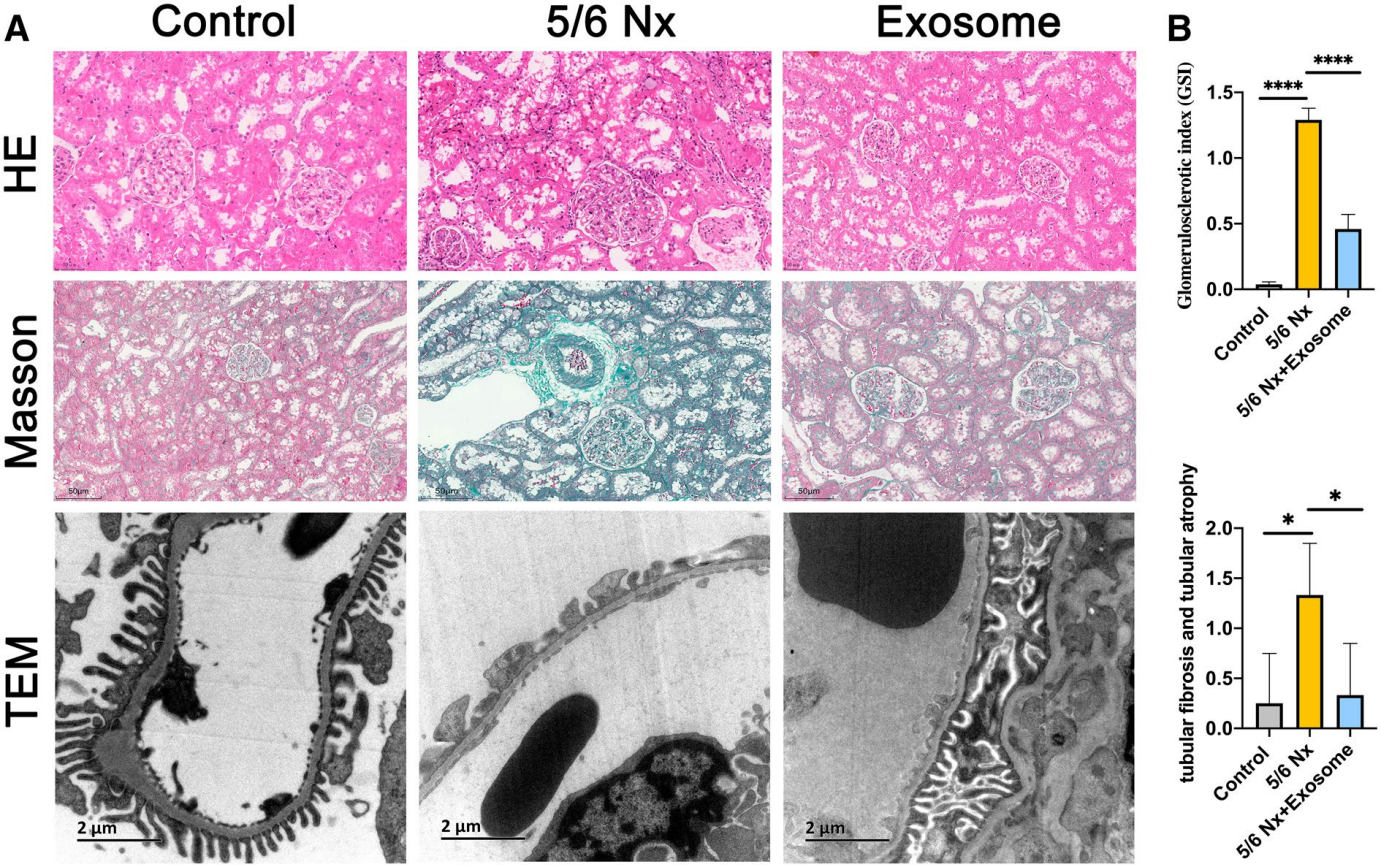
Effect of exosomes on renal pathology. **A** Representative images of H & E, and Masson’s staining and TEM from control, 5/6 Nx, and exosome-treated 5/6 Nx rats. **B** Glomerular sclerosis and tubular fibrosis and tubular atrophy were evaluated using a semi-quantitative scoring method. *p<0.05, ****p<0.0001

**Fig. 4:**
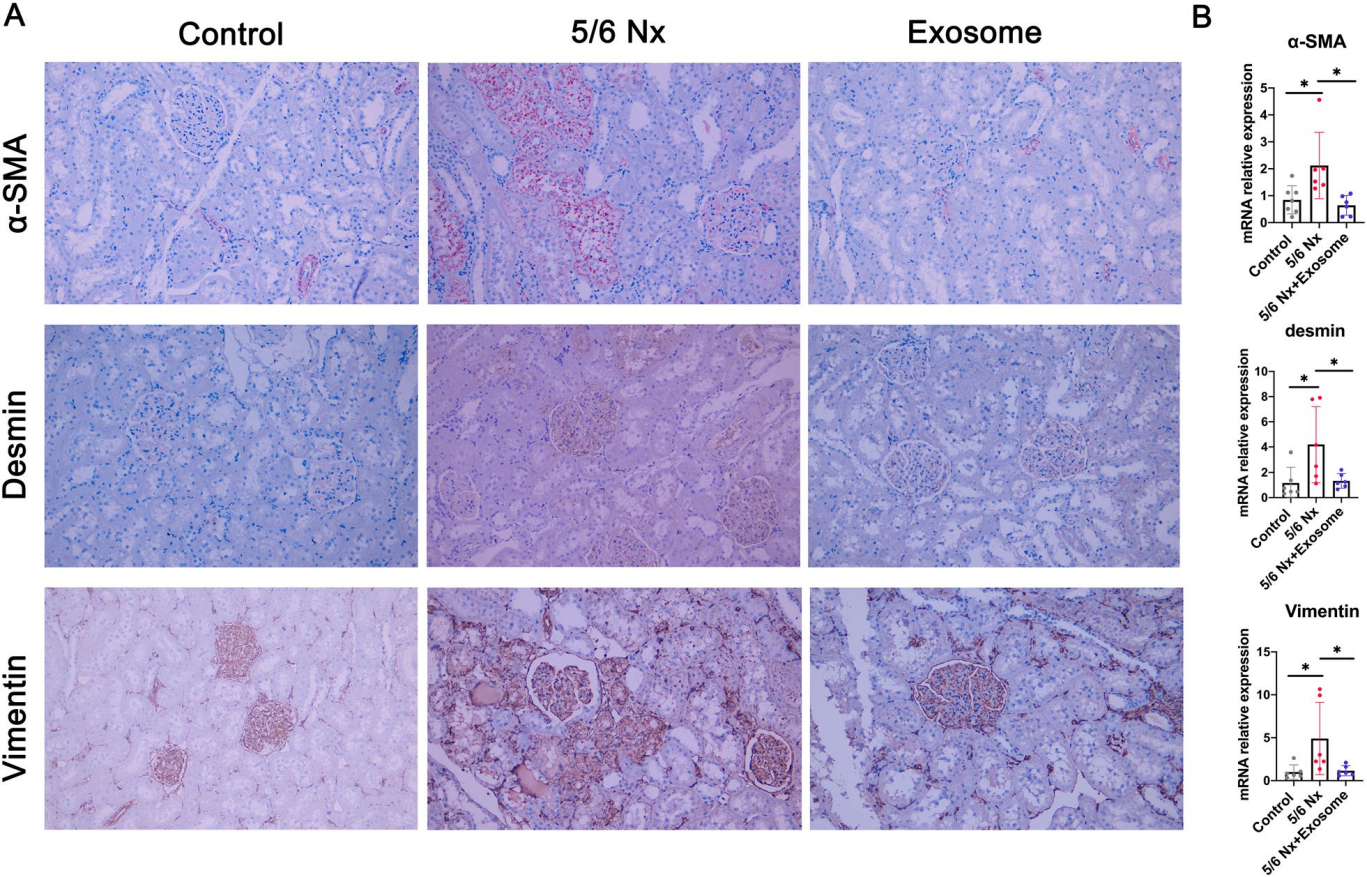
Detection of the expression of α-SMA, desmin, and vimentin. **A** Immunohistochemical staining of α-SMA, desmin, and vimentin. **B** mRNA expression of α-SMA, desmin, and vimentin in rat kidney tissue. Data are shown as the mean ± SD. *p<0.05

As shown in Fig. 3, control rats had well-opened glomerular capillary loops, closely arranged renal tubules, and an intact foot process. In contrast, 5/6 Nx rats exhibited glomerulosclerosis, mesangial cell proliferation, interstitial fibrosis, and inflammatory cell infiltration in the renal interstitium, partial fusion, and flattening of the foot process (Fig. 3). However, these lesions were relieved upon exosome treatment. In addition, immunochemical staining results showed that the expression levels of fibrotic markers such as α-SMA, desmin, and vimentin were significantly increased in the renal tubule region in 5/6 Nx rat (Fig. 4A). However, their expression levels were significantly decreased in the exosome treatment group. Similar results were observed at the mRNA level as evaluated by RT-qPCR (Fig. 4B). These results indicated that rBMSC-derived exosomes improved renal pathological injury in 5/6 Nx rats.

### rBMSCs-derived exosome could regulate klotho expression

**Fig. 5:**
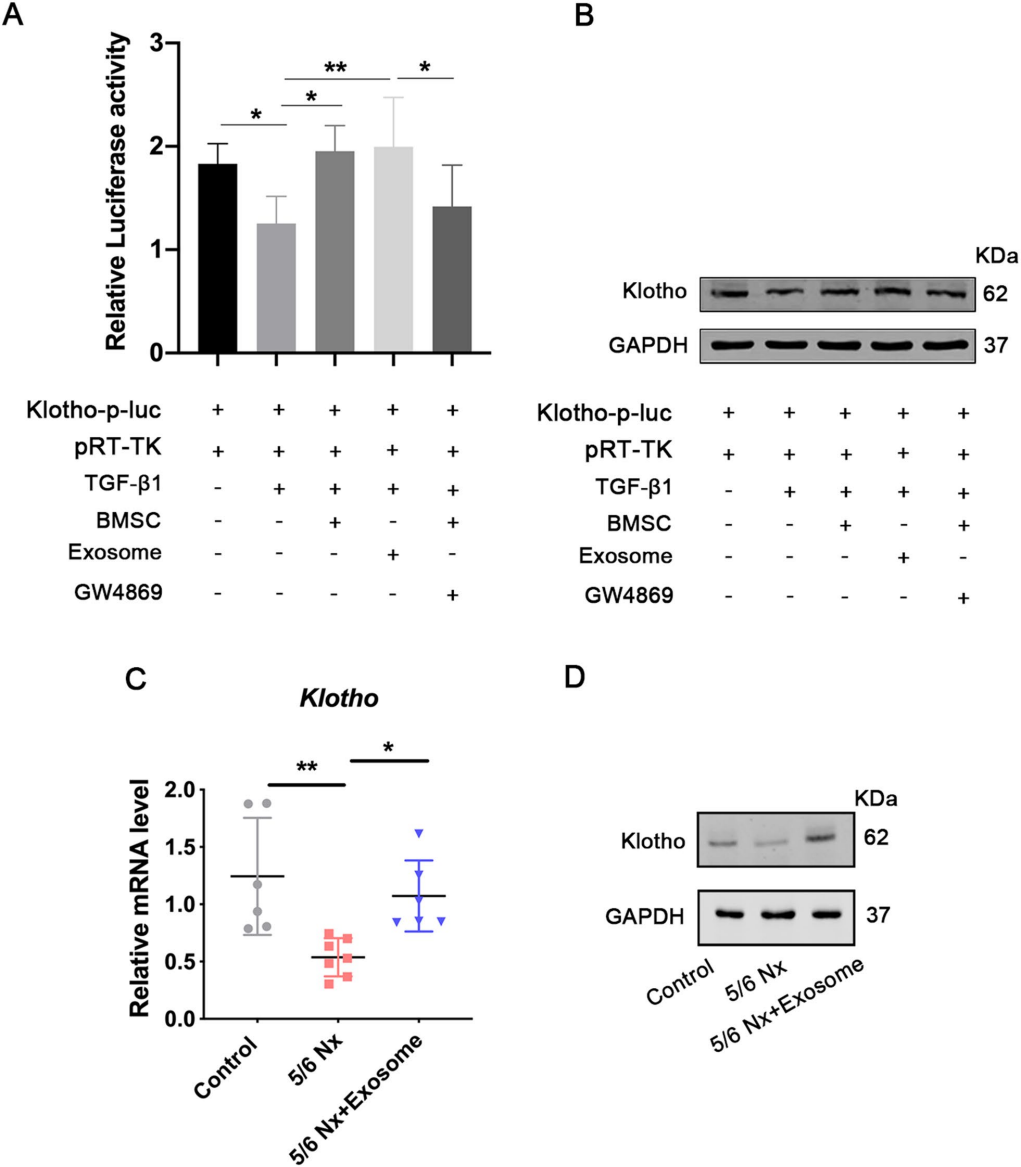
Effect of exosome transplantation on klotho activation and expression. **A** Fluorescent report plasmid containing klotho promoter and pRL-TK were co-transfected into 293 T cells. The transfected cells were randomly divided into five groups: control group (PBS), TGF-β1 group (1 ng/μL TGF-β1), BMSCs group (1 ng/μL TGF-β1+co-cultured with 5 × 10^4^ well BMSCs), exosomes (1 ng/μL TGF-β1+0.4 mg exosomes), and exosome inhibitor group (1 ng/μL TGF-β1+co-cultured with 5 × 10^4^ well BMSCs+GW4869). Then the firefly and Renilla luciferase activities were measured using the dual luciferase reporter gene assay. Data are shown as mean ± SD (*n* = 3). *p < 0.05, **p < 0.01. The protein expression of klotho was also evaluated using western blotting **B**, **C** qRT-PCR were performed to detect the mRNA expression of klotho. mRNA expression levels were normalized to that of GAPDH. Quantitative data (*n* = 6–7) are provided as the mean ± SD. *p < 0.05, **p < 0.01. **D** Expression of klotho protein in the control, 5/6 Nx, and exosome-treated 5/6 Nx rats was evaluated using western blotting

To evaluate the effect of exosomes on klotho activation, 293 T cells were co-transfected with the klotho-luc reporter and pRL-TK, which encoded Renilla luciferase. The transfected cells were treated with TGF-β1 for 24 h. Results showed that TGF-β1 inhibited klotho activity; however, the activity was increased by both BMSC co-culture and exosome treatment group (Fig. 5A). In addition, klotho activity did not increase in the cells treated with both BMSC and exosome biogenesis/release inhibitor group (Fig. 5A). These results suggest that rBMSCs-derived exosomes could regulate the klotho promoter. Similar results were observed at the protein level (Fig. 5B). In addition, the mRNA and protein expression levels of klotho were markedly decreased in the kidneys of 5/6 rats, whereas their expression levels were upregulated in the rBMSC-derived exosome-treated group (Fig. 5C, D). Collectively, these results indicate that rBMSC-derived exosomes may regulate the activity and expression of klotho.

## Discussion

In this study, we explored the effect of rBMSC-derived exosomes on kidney injury in 5/6 Nx rats. The 5/6 Nx rats treated with exosomes exhibited significantly decreased proteinuria, improved renal function, and inhibited renal pathological features. Further experiment showed that exosomes could upregulate klotho expression by regulating the promoter activity of klotho. Our study outcomes add new information to this research field, thereby providing a theoretical basis for clinical treatment of CKD in the future.

MSCs have been widely explored in experimental models of CKD [14]. In mice with Streptozotocin (STZ)-induced type 1 diabetes, MSC transplantation prevented kidney injury and decreased albuminuria significantly [15]. Another study showed that BMSCs exerted protective effects in rats with diabetic nephropathy by reducing the number and inhibiting maturation of CD103^+^ DCs, thus regulating the CD8 T cell responses [5]. Human umbilical cord mesenchymal stem cells also improved the renal function and prevented the progression of STZ-induced diabetic nephropathy in rats [16]. Amnion-derived mesenchymal stem cells were reported to ameliorate CKD in a 5/6 Nx rat model [17]. Consistently, we previously reported that BMSCs attenuated the progression of renal damage in FSGS rats [10]. Collectively, these findings suggest that MSCs therapeutic approach is effective in animal studies. Interestingly, in our previous study, we observed that a significant number of fluorescence-labeled MSCs, injected via the caudal vein, did not reach the kidneys but were lodged in the lungs instead. These results indicated that the beneficial effects of MSCs may be mainly attributed to the paracrine mechanisms.

However, several challenges need to be overcome before MSCs can be applied for the treatment of kidney diseases. The technical difficulty in directly injecting BMSCs into the renal parenchyma or subcapsular space to exert their therapeutic effects is one of these challenges. Furthermore, biosafety remains a pertinent issue as transplanted cells may increase the risk of generating neoplasms. Thus, in terms of controlling and reducing risk, MSC-derived exosomes are promising alternative agents for treating kidney diseases.

As an “aging-suppressor” gene, klotho expression exhibits tissue specificity and is highly expressed in renal tubules [18]. Existing evidence reveals that abnormal klotho levels are regarded as the earliest biochemical abnormality of kidney disease [19–22]. Therefore, klotho is considered a key target for the treatment of kidney disease. Currently, the regulatory mechanism of klotho is complicated and under intensive investigation. Rodrigues et al. reported human umbilical cord-derived mesenchymal stromal cells treatment upregulated klotho protein expression in ischaemia/reperfusion injury-induced renal senescence in rats [23]. We found BMSC could also upregulate klotho expression in our preliminary experiment (data not shown). Studies showed MSC-derived exosomes contain many proteins, microRNAs, mRNAs, long non-coding RNAs, and phospholipids [24, 25]. Mehi et al. identified that there were many microRNA binding sites within the 3′ untranslated region of klotho gene, such as microRNA-339, microRNA-556, microRNA-10, and microRNA-199 [26]. Similarly, many studies found other microRNAs could also regulate klotho [27–29]. We, therefore, hypothesized that microRNAs in exosomes might play an important role in regulating klotho. Here we found rBMSC-derived exosomes could ameliorate kidney function of 5/6 Nx rats, and the effect possibly was related to the modulation of klotho expression. However, the limit of this study is the lack of klotho knockout rat in our study to further validate the causal relationship of exosomes and klotho. We will continue to explore the accurate molecular mechanism in our further research.

In conclusion, we demonstrated that BMSCs-derived exosomes alleviated renal injury in 5/6 Nx rats probably by upregulating klotho activity and expression. These results provide new insight into stem cell therapy for nephropathy.

## Supplementary Information

**Additional file 1. The western blot membrane for CD63 in Figure 1B.**

**Additional file 2**. **The western blot membrane for CD9 in Figure 1B.**

**Additional file 3**.** The western blot membrane for β-actin in Figure 1B.**

**Additional file 4**. **The western blot membrane for GAPDH in Figure 5B.**

**Additional file 5**. **The western blot membrane for klotho in Figure 5B.**

**Additional file 6**. **The western blot membranes for klotho and GAPDH in Figure 5D.**

## Data Availability

The data sets generated and analyzed during the current study are available from the corresponding author on reasonable request.

## Abbreviations

MSCs: Mesenchymal stem cells
rBMSCs: Rat bone marrow mesenchymal stem cells
Nx: Nephrectomy
CKD: Chronic kidney disease
SCr: Serum creatinine
BUN: Blood urea nitrogen
NTA: Nanoparticle tracking analysis
STZ: Streptozotocin
