# Supplementary figures and images for: BMSC-derived exosomes protect against kidney injury through regulating klotho in 5/6 nephrectomy rats
Source: Eur J Med Res. 2022 Jul 11;27:118. doi: 10.1186/s40001-022-00742-8 (PMC9277829; doi:10.1186/s40001-022-00742-8)

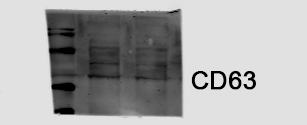

Supplement: Supplementary file 1 — Additional file 1. The western blot membrane for CD63 in Figure 1B. [file 40001_2022_742_MOESM1_ESM.tif]

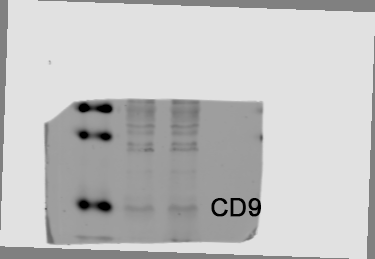

Supplement: Supplementary file 2 — Additional file 2. The western blot membrane for CD9 in Figure 1B. [file 40001_2022_742_MOESM2_ESM.tif]

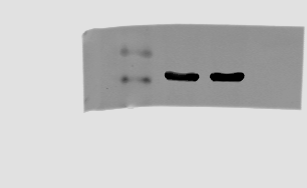

Supplement: Supplementary file 3 — Additional file 3. The western blot membrane for β-actin in Figure 1B. [file 40001_2022_742_MOESM3_ESM.tif]

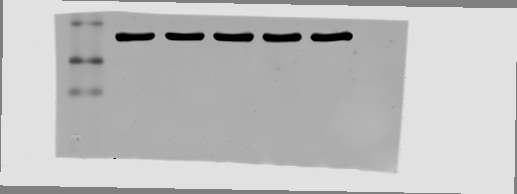

Supplement: Supplementary file 4 — Additional file 4. The western blot membrane for GAPDH in Figure 5B. [file 40001_2022_742_MOESM4_ESM.tif]

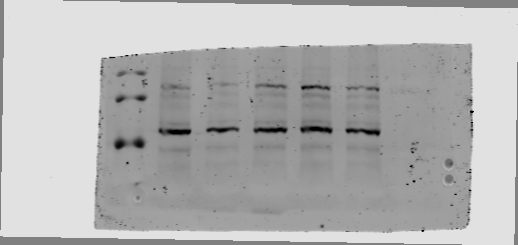

Supplement: Supplementary file 5 — Additional file 5. The western blot membrane for klotho in Figure 5B. [file 40001_2022_742_MOESM5_ESM.tif]

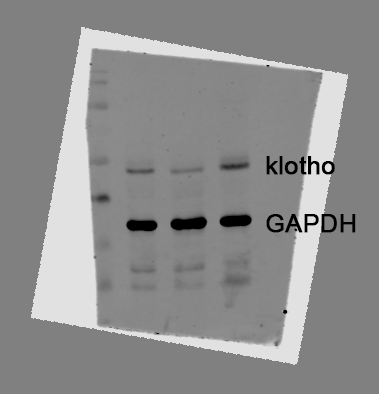

Supplement: Supplementary file 6 — Additional file 6. The western blot membranes for klotho and GAPDH in Figure 5D. [file 40001_2022_742_MOESM6_ESM.tif]
